# Taurine reduces microglia activation in the brain of aged senescence-accelerated mice by increasing the level of TREM2

**DOI:** 10.1038/s41598-024-57973-4

**Published:** 2024-03-28

**Authors:** Sharif Ahmed, Ning Ma, Jun Kawanokuchi, Keiya Matsuoka, Shinji Oikawa, Hatasu Kobayashi, Yusuke Hiraku, Mariko Murata

**Affiliations:** 1 Department of Environmental and Molecular Medicine, Mie University Graduate School of Medicine, 2-174 Edobashi, Tsu, Mie 514-8507 Japan; 2 Department of Environmental Health, University of Fukui School of Medical Sciences, Eiheiji, Fukui Japan; 3 Graduate School of Health Science, Suzuka University of Medical Science, Suzuka, Mie Japan; 4 Institute of Traditional Chinese Medicine, Suzuka University of Medical Science, Suzuka, Mie Japan; 5 Department of Acupuncture and Moxibution Science, Faculty of Health Science, Suzuka University of Medical Science, Suzuka, Mie Japan

**Keywords:** Neuroscience, Medical research

## Abstract

Alzheimer’s disease (AD), a chronic neurodegenerative disorder, is the leading cause of dementia. Over-activated microglia is related to amyloid-beta (Aβ) and phosphorylated tau (phospho-tau) accumulation in the AD brain. Taurine is an amino acid with multiple physiological functions including anti-inflammatory effects, and has been reported to be neuroprotective in AD. However, the role of taurine in microglia-mediated AD remains unclear. Here, we examined the effects of taurine on the brains of senescence-accelerated mouse prone 8 (SAMP8) mice by comparing those administered 1% taurine water with those administered distilled water (DW). We observed increased levels of taurine and taurine transporter (TAUT) in the brains of the taurine-treated mice compared with those of control mice. Immunohistochemical and Western blot analyses revealed that taurine significantly reduced the number of activated microglia, levels of phospho-tau and Aβ deposit in the hippocampus and cortex. Triggering receptors expressed on myeloid cells-2 (TREM2) are known to protect against AD pathogenesis. Taurine upregulated TREM2 expression in the hippocampus and cortex. In conclusion, the present study suggests that taurine treatment may upregulate TREM2 to protect against microglia over-activation by decreasing the accumulation of phospho-tau and Aβ; providing an insight into a novel preventive strategy in AD.

## Introduction

Alzheimer's disease (AD) is the most common and well-known cause of dementia, and is characterized by progressive deficits in memory and cognitive abilities. In people aged ≥ 65 years, cognitive dysfunction, language impairment, memory loss and other neurodegenerative disorders are usually observed over time^1^. AD brain pathology is characterized by the deposition of senile plaques consisting of extracellular amyloid-beta (Aβ) and neurofibrillary tangles within neurons composed of phosphorylated tau (phospho-tau)^2,3^. Aβ, a protein that exists in several different molecular forms, is formed when a large transmembrane amyloid precursor protein is cleaved in the cortex and hippocampus^3^. Tau is a microtubule-associated protein involved in the stabilization of neuronal microtubules in the axons^4^. Microglia are resident macrophages of the central nervous system (CNS) and play an important role in repair and defense mechanisms, such as removing dead cells, cellular debris and other harmful materials by phagocytosis in the brain^5,6^. However, there is abundant evidence that activated microglia can be harmful to neurons, leading to neurodegenerative disorders such as AD^7^. Several studies have reported a relationship between Aβ and tau hyperphosphorylation in AD pathology. Busche and Hyman^8^ indicate synergy between Aβ and tau in AD to elucidate disease pathogenesis. Zhang et al.^9^ also suggest reciprocal toxicity between Aβ and tau, and combined effects of Aβ and tau on microglia activation. These studies imply that combined therapies against Aβ and tau may be the most effective way to improve microglia status and neuronal function.

In the brain, the triggering receptor expressed on myeloid cells-2 (TREM2) is predominantly expressed in microglia, and TREM2 controls many important functions of microglia, including phagocytosis of tissue debris^10,11^. Its loss-of-function variants and impaired TREM2 function have been identified as risk factors for neurodegenerative diseases including AD^10,12^. Ulland et al. found that microglia in AD patients carrying TREM2 risk variants, indicating that TREM2 regulates microglial function through modulation of cellular biosynthetic metabolism^13^. Several studies have focused on the impact of TREM2 on Aβ and tau pathology^14,15^, and their findings suggest a relation between them, which may be a plausible therapeutic target for AD.

Taurine (2-aminoethanesulfonic acid), a sulfur-containing semi-essential amino acid, is one of the most abundant amino acids found in mammals. All the physiological roles that have been found for taurine are crucial in the developmental process, such as improvement of blood pressure, neuroprotective function, anti-inflammation, anti-oxidation and calcium homeostasis^16–19^. Several reports have indicated that taurine can ameliorate various neurological disorders^20,21^. 5XFAD mice, 3xTg mice, and APP/PS1 mice are widely used as Alzheimer’s disease model mice^22,23^. Orally administered taurine with drinking water can ameliorate cognitive impairment by directly binding to oligomeric Aβ in APP/PS1 transgenic mice^24^. Senescence-accelerated mouse prone 8 (SAMP8) has been established as a neuropathological model of accelerated brain aging and dementia^25^, and its phenotypes resemble those of patients with late-onset and age-related sporadic AD^26^. However, there have been no reports on the effects of taurine in SAMP8 mice.

Therefore, the present study aims to examine the effect of taurine by administering 1% taurine water to SAMP8 mice from 20 to 42 weeks-old, as microglia in the hippocampus and cortex may be activated due to the deposition of phosphorylated tau (phospho-tau) and Aβ, which are reported to appear at 5 and 8 months old, respectively^27–29^. AD dysregulation of Aβ and tau metabolism progressively disrupt normal synaptic function, leading to hippocampal atrophy. The hippocampus has a central role in semantic and episodic memory processing, and this cognitive function is critically dependent on hippocampal functional connectivity with many cortical regions, including temporal cortex^30^. Therefore, we examine both hippocampus and temporal cortex in this study. Additionally, we evaluated the expression of TREM2 in brain tissues to determine the impact of TREM2 on Aβ and tau pathology.

## Results

### Taurine treatment increases the levels of taurine and taurine transporter in SAMP8 mouse hippocampus and cortex

**Figure 1:**
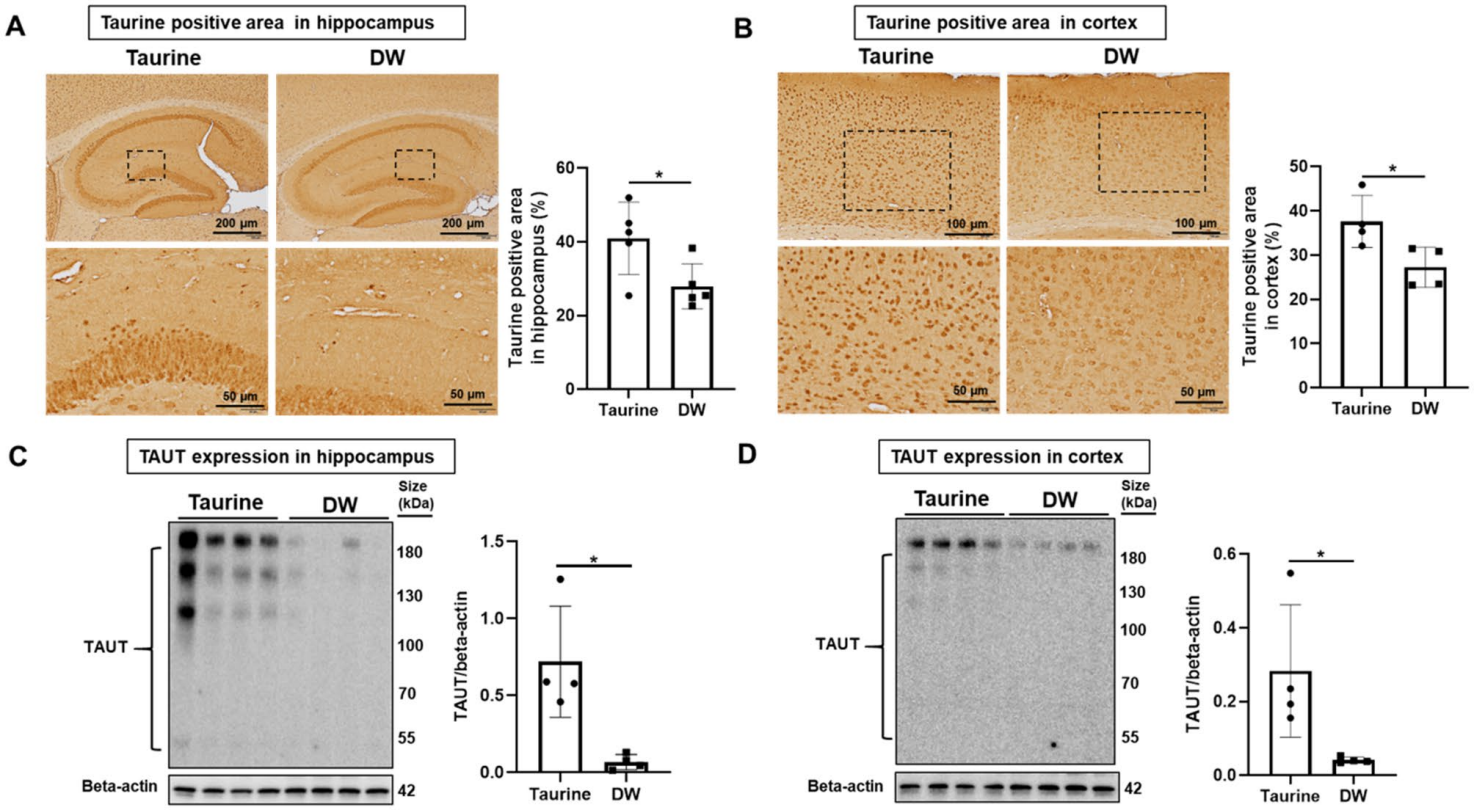
Effects of taurine treatment on taurine-positive area and TAUT in brain tissue. Representative immunohistochemical staining images of taurine and quantitative analyses of taurine-positive area in the hippocampus (**A**, top row, bar = 200 µm; bottom row, bar = 50 µm, enlarged from the dashed line box) and cortex (**B**, top row, bar = 100 µm; bottom row, bar = 50 µm, enlarged from the dashed line box). Taurine-positive areas are visualized in brown. Data are expressed as the means ± SD of 5 hippocampal fields from five SAMP8 mice and 4 specific cortex areas from four SAMP8 mice of each group. Western blots of TAUT expression in the hippocampus (**C**) and cortex (**D**). Data are expressed as the means ± SD of quadruplicate samples of each group. *p < 0.05, Student’s *t*-test.

In this study, we treated SAMP8 mice with 1% taurine in drinking water for 22 weeks until sacrifice at 42 weeks-old. We checked the levels of taurine and taurine transporter (TAUT) in the brain tissues of SAMP8 mice. Immunohistochemical analyses showed that taurine group significantly increased the taurine-positive area in the hippocampus (Fig. 1A) and cortex (Fig. 1B) compared with the distilled water (DW) group. Western blot analyses showed that taurine group had an increased level of TAUT expression in the hippocampus (Fig. 1C) and cortex (Fig. 1D) compared with DW group.

### Taurine treatment reduces the number of activated microglia in SAMP8 mouse hippocampus and cortex

**Figure 2:**
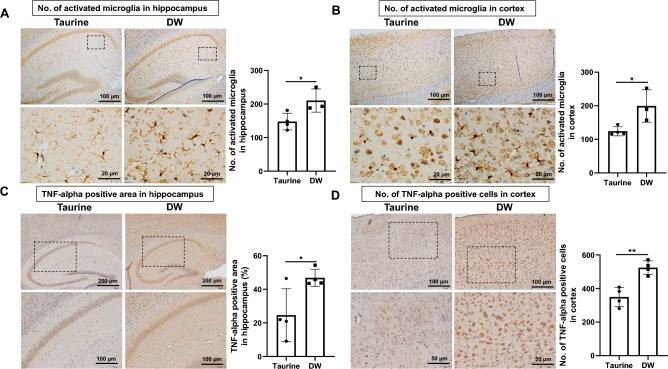
Effects of taurine treatment on microglial cells in brain tissue. Representative immunohistochemical staining images of Iba-1 (microglia stained in brown) showing microglial activation with quantitative analyses of activated microglia in the hipppocampus (**A**, top row, bar = 100 µm; bottom row, bar = 20 µm, enlarged from the dashed line box) and cortex (**B**, top row, bar = 100 µm; bottom row, bar = 20 µm, enlarged from the dashed line box). Nuclei were counterstained with hematoxylin. Quantification of the immunohistochemistry data are expressed as the means ± SD of 3–4 hippocampal fields from three to four SAMP8 mice and 3–4 specific cortex areas from three to four SAMP8 mice of each group. Representative immunohistochemical staining images of TNF-alpha positive area in hippocampus (**C**, top row, bar = 200 µm; bottom row, bar = 100 µm, enlarged from the dashed line box) and TNF-alpha positive cells in cortex (**D**, top row, bar = 100 µm; bottom row, bar = 50 µm, enlarged from the dashed line box). Nuclei were counterstained with hematoxylin. Quantification of the immunohistochemistry data are expressed as the means ± SD of 4 hippocampal fields from four SAMP8 mice and 4 specific cortex areas from four SAMP8 mice of each group. *p < 0.05 and **p < 0.01 by Student’s *t*-test.

Microglia plays an important role in AD pathogenesis. The tissue sections were stained with an Iba-1 antibody to detect microglia^31,32^. Immunohistochemical analysis confirmed Iba-1 staining in the microglia of SAMP8 mice. Visual inspection and manual counting confirmed that the number of activated microglia in the hippocampus (Fig. 2A, top row) and cortex (Fig. 2B, top row) of the taurine group was significantly lower than that in the DW group. The patterns of typical activated microglia in the hippocampus and cortex are shown in the bottom row of Fig. 2A and B respectively.

We performed IHC analyses of TNF-alpha to show the broader impact of taurine treatment on neuroinflammation in SAMP8 mice. TNF-alpha positive area was significantly lower in the hippocampus of taurine group than that in the DW group and number of TNF-alpha positive cells in cortex was significantly lower in the taurine group than that in the DW group, shown in Fig. 2C (hippocampus) and Fig. 2D (cortex).

### Taurine treatment reduces the accumulation of phospho-tau in SAMP8 mouse hippocampus and cortex

**Figure 3:**
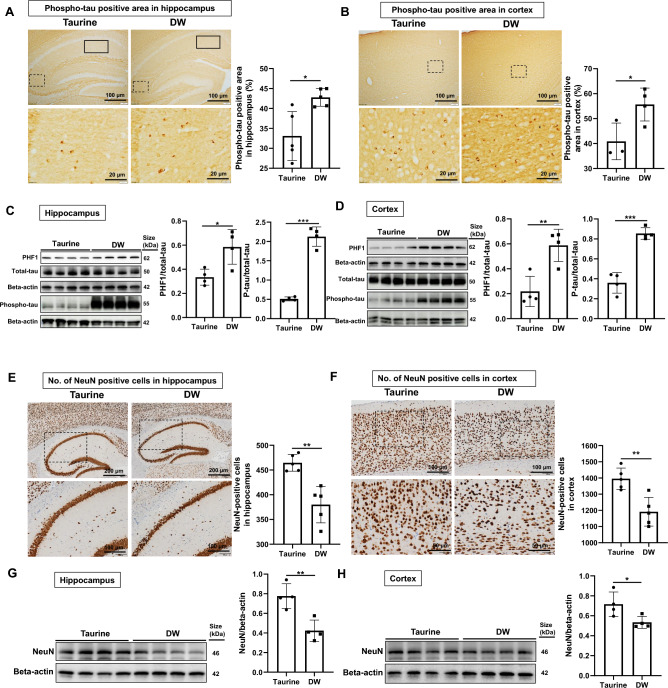
Effects of taurine treatment on tau hyperphosphorylation. Representative immunohistochemical staining images of phospho-tau positive area (brown) in the hippocampus (**A**, top row, bar = 100 µm; bottom row, bar = 20 µm, enlarged from the dashed line box) and cortex (**B**, top row, bar = 100 µm; bottom row, bar = 20 µm, enlarged from the dashed line box). The rectangular boxes in Fig. 3A indictae the CA1 hippocampal area where phospho-tau was analyzed. Data are expressed as the means ± SD of 3–5 hippocampal fields from three to five SAMP8 mice and 3–5 specific cortex areas from three to five SAMP8 mice of each group. Western blots of PHF1, phospho-tau and total-tau protein levels, bands observed depict the quantitative analyses of PHF1 phospho-tau and total-tau in the hippocampus (**C**) and cortex (**D**). Quantification of Western blot data are expressed as the means ± SD of quadruplicate samples of each group. Representative immunohistochemical staining images shows NeuN positive cells in hippocampus (**E**, top row, bar = 200 µm; bottom row, bar = 100 µm, enlarged from the dashed line box) and cortex (**F**, top row, bar = 100 µm; bottom row, bar = 50 µm, enlarged from the dashed line box). Data are expressed as the means ± SD of 5 hippocampal fields from five SAMP8 mice and 5 specific cortex areas from five SAMP8 mice of each group. Western blots of NeuN expression in the hippocampus (**G**) and cortex (**H**). The values presented are the means ± SD of quadruplicate samples of each group. *p < 0.05, **p < 0.01 and ***p < 0.001 by Student’s *t*-test.

Since several studies have suggested that over-activated microglia increase the deposition of phospho-tau in the CNS^33^, we examined the accumulation of phospho-tau in the hippocampal and cortical tissue after taurine treatment in SAMP8 mice. Immunohistochemical analyses showed accumulation of phospho-tau in the hippocampus and cortex. In the hippocampus, we observed lower accumulation of phospho-tau (Fig. 3A) in the taurine group than in the DW group. In the cortex (Fig. 3B), the phospho-tau-positive areas were significantly decreased by taurine treatment. Additionally, we confirmed by Western blotting that taurine group significantly lowers the accumulation of phospho-tau and PHF1 in SAMP8 mice hippocampus (Fig. 3C) and cortex (Fig. 3D) compared with DW group. Furthermore, we performed IHC and WB analyses with a neuronal marker, neuronal-nuclei (NeuN). IHC analyses shows that taurine treatment significantly reduced the neural loss in both hippocampus (Fig. 3E) and cortex (Fig. 3F), and WB analyses shows similar results in both hippocampus (Fig. 3G) and cortex (Fig. 3H).

### Taurine treatment reduces the accumulation of Aβ in SAMP8 mouse hippocampus and cortex

**Figure 4:**
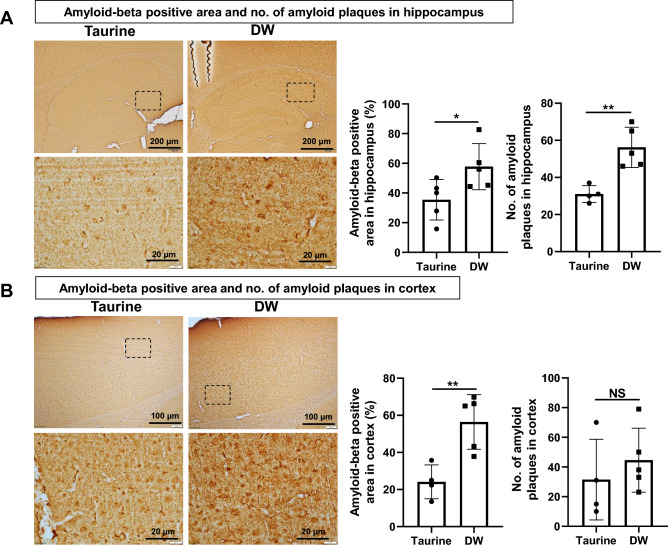
Effects of taurine treatment on Aβ accumulation in brain tissue. Representative immunohistochemical staining images of Aβ positive area (brown), its quantitative analyses and deposition of amyloid plaques in the hippocampus (**A**, top row, bar = 200 µm; bottom row, bar = 20 µm, enlarged from the dashed line box) and cortex (**B**, top row, bar = 100 µm; bottom row, bar = 20 µm, enlarged from the dashed line box). Data are expressed as the means ± SD of 4–5 hippocampal fields from four to five SAMP8 mice and 4–5 specific cortex areas from four to five SAMP8 mice of each group. *p < 0.05 and **p < 0.01, Student’s *t*-test.

Since over-activated microglia has been reported to be involved in the deposition of Aβ in the CNS^33^, we examined the deposition area of Aβ and number of amyloid plaques in the SAMP8 mice. Both intra- and extracellular Aβ deposits were observed. Relevantly, LaFeria et al. indicated that intracellular Aβ deposit may contribute to AD progression^34^. Immunohistochemical analyses revealed that Aβ-positive area was significantly smaller and the number of amyloid plaques in the hippocampus was significantly lower (Fig. 4A) in the taurine group than in the DW group. In the cortex, when compared with the DW group, the taurine group had significantly smaller Aβ-positive area, although no significant difference in the number of amyloid plaques (Fig. 4B) was observed. The patterns of amyloid plaques in the hippocampus and cortex are shown in the bottom rows of Fig. 4A and B respectively.

### Taurine treatment upregulates TREM2 in SAMP8 mouse hippocampus and cortex

**Figure 5:**
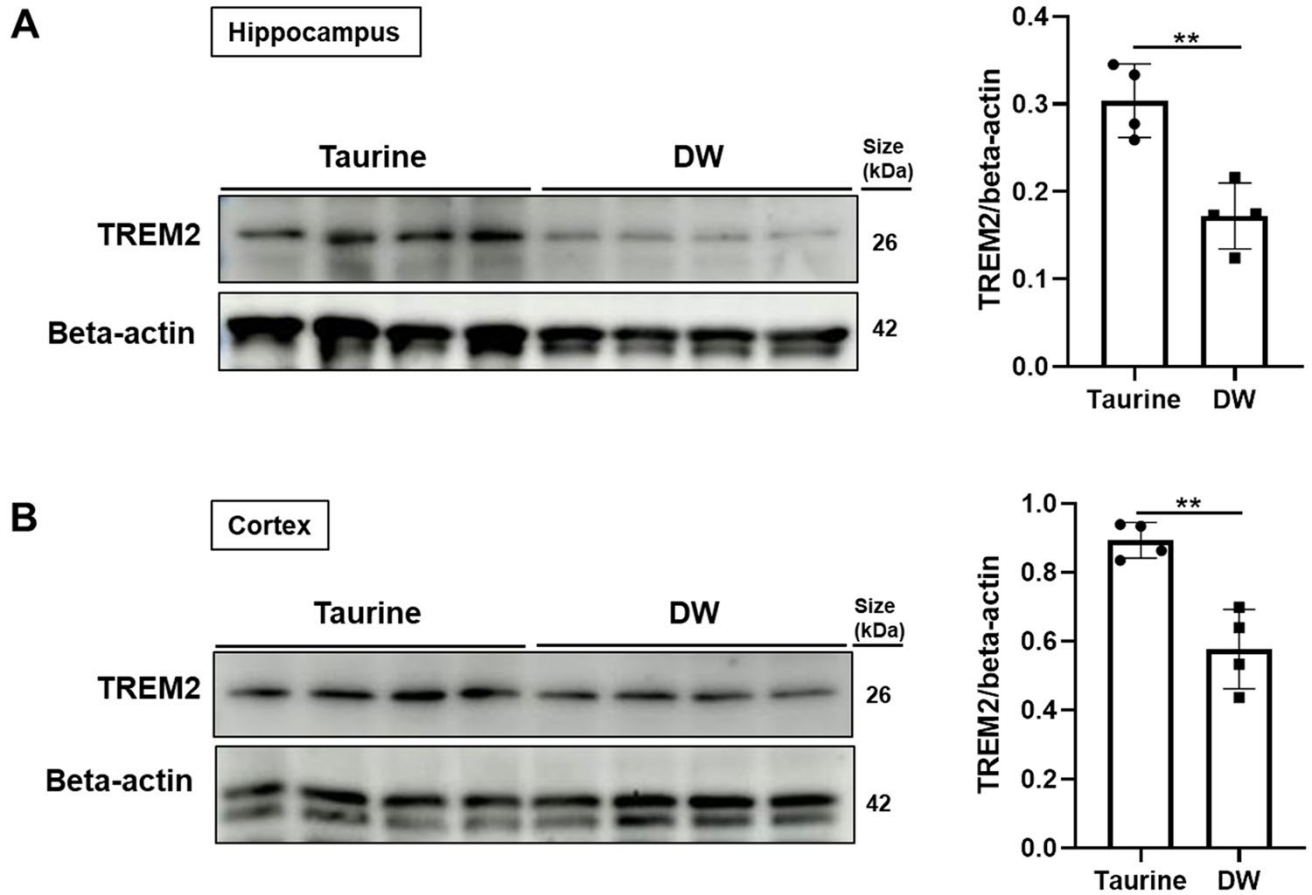
Effects of taurine treatment on TREM2 in brain tissue. Expression of TREM2 were analyzed by Western blotting. Quantitative analyses of TREM2 level in the hippocampus (**A**) and cortex (**B**). Quantification of Western blot data is expressed as the means ± SD of quadruplicate samples of each group. **p < 0.01, Student’s *t*-test.

TREM2 is a cell surface receptor that is mainly abundant in microglia. Many researchers have reported that overexpression of TREM2 lowered the accumulation of phospho-tau^35,36^ and Aβ^37^ in AD. To elucidate a mechanism by which taurine lowers the accumulation of phospho-tau and Aβ deposition, we examined the levels of TREM2 in the hippocampus and cortex of SAMP8 mice by Western blotting. Western blot analyses showed that taurine group significantly increased the expression of TREM2 in the hippocampus (Fig. 5A) and cortex (Fig. 5B) compared with DW group.

## Discussion

AD hallmarks are Aβ extracellular deposits, intracellular hyperphosphorylated tau aggregates, and pathologically activated microglia, which drives neurodegeneration and atrophy within the cortex and hippocampus^38^. The hippocampus and cerebral cortex are involved in cognitive function and neurogenesis in the brain. SAMP8 mice exhibit many characteristics of AD pathogenesis, including abnormal expression of anti-aging factors, deposition of Aβ and hyperphosphorylation of tau. In the present study, we demonstrated the protective effects of taurine against Aβ and phospho-tau deposits with microglia activation in SAMP8 mice. We confirmed that taurine treatment significantly increased the amount of taurine and TAUT expression in the hippocampus and cortex. Our group recently reported that taurine treatment increased TAUT expression, leading to higher taurine levels in tumor tissues in vivo, which exhibited an anti-tumor effect^39^. Based on these findings, we hypothesized that taurine may exert neuroprotective effects by reducing the number of activated microglia in the hippocampus and cortex. Under normal physiological conditions, the microglia exhibit limited cytokine expression and no phagocytic activity. Due to the response of various harmful stimuli including Aβ and phospho-tau deposits, microglia exhibit phagocytic function and overexpresses cytokine modulators, which is known as microglia activation^38^. Microglial activation is a key neuropathological feature of AD. In this study, we found that taurine treatment significantly reduces the expression of phospho-tau and Aβ in the hippocampal and cortical tissues of SAMP8 mice. Javed et al.^40^ reported that taurine ameliorated neurobehavioral changes and protected the morphology of hippocampal pyramidal neurons in sporadic Alzheimer’s type dementia caused by streptozotocin in rats. Taurine treatment has been reported to rescue cognitive deficits in a transgenic mouse model of AD, with slight decrease of Aβ insoluble fraction^24^. Glutamate PET imaging showed the neuroprotective effect of taurine in an AD transgenic mice model, wherein brain uptake improved with taurine treatment; however, taurine had no effect on Aβ pathology when observed under the similar conditions^21^. Moreover, taurine decreases phosphorylated tau protein levels in the brains of AD rats^41^. Few studies have demostrated the therapeutic potential of taurine for neurological disorders^42,43^. These findings are consistent with our results and support the hypothesis of taurine-mediated neuroprotection.

TREM2 is a transmembrane receptor that is widely expressed by microglia in the brain and is involved in the neuroinflammatory responses in AD. Takahashi et al.^44^ reported that TREM2 knockdown in microglia inhibited the phagocytosis of apoptotic neurons, whereas TREM2 overexpression increased phagocytosis and decreased microglial proinflammatory responses. Ruganzu et al.^45^ reported that TREM2 overexpression rescued cognitive deficits, reduced amyloid plaque deposition and improved synaptic function in APP/PS1 transgenic mice. In this study, we analyzed the TREM2 levels in the mouse brain. We first demonstrated that taurine treatment significantly increased the expression of TREM2 compared with DW treatment in SAMP8 mice, potentially leading to a reduction in microglia activation and accumulation of Aβ and phospho-tau. Several studies have found that Aβ and phosphorylated tau accumulation significantly increased with age in SAMP8 mice compared with SAM resistant 1 control model^46^. TREM2 expression is modulated by inflammation and upregulation of TREM2 might induce the downregulation of some proinflammatory factors, such as IL-1β and TNF-α^47^. As a microglial surface receptor, TREM2 interacts with the adaptor protein DAP12 (TYRO protein tyrosine kinase-binding protein TYROBP) to initiate signal transduction pathways that promote microglial cell activation, phagocytosis and microglial survival^48^. Peng et al.^14^ recently reported that upregulation of TREM2 inhibits tau hyperphosphorylation and neuronal apoptosis via the activation of PI3K/Akt/GSK-3β signaling pathway where phosphorylation of Akt was decreased in the brain of APP/PS1 mice. Our group previously reported that taurine exhibits an apoptosis-inducing effect on human nasopharyngeal carcinoma cells through the PTEN/AkT pathway, where taurine treatment significantly reduces the phosphorylation of Akt^49^.

There are several limitations. In this study, we did not perform the behavioral experiments to assess whether the observed reductions in Aβ and phospho-tau levels correlate with improvements in cognitive functions or memory in the treated mice. We examined SAMP8, alone. Further studies are needed to clarify the relationship between cognitive improvement and the levels of Aβ and phospho-tau in taurine-treated wild type, SAPM8, and other AD models, such as 5XFAD, 3xTg, and APP/PS1 mice. While the exact mechanism of taurine-induced protective effect in senescent-accelerated mouse brain has not yet been ascertained, our findings indicate that taurine has therapeutic potential against AD by attenuating Aβ and tau pathology via TREM2-mediated signaling pathways and that these molecules may be preventive and therapeutic targets of AD.

## Methods

### Animals

Four-week-old male senescence-accelerated mice (SAMP8) were purchased from Japan SLC (Shizuoka, Japan). Mice were maintained in a single cage (one mouse per cage) with wood-derived bedding materials under standard housing conditions (temperature: 21 ± 1 °C; humidity: 55 ± 2%) in a specific pathogen free facility with a 12 h light/dark cycle and given food ad libitum. All experimental protocols for the present study were approved by the Suzuka University Medical Science Board Committee for Animal Care and Use of Laboratory Animals (Approval No. 259). All the methods in the study were performed as per the guideline for the laboratory animal facilities. The reporting of the studies was done in accordance with ARRIVE guidelines.

### Experimental protocol

Mice were randomly divided into two groups at 20 weeks of age. One group was administered water with 1% taurine, the taurine group, while the mice in the other group were administered DW, the DW group. It is estimated that mice ingest 20 mg/day of taurine based on their water intake. Body weights were measured monthly. To observe their health status, we started with twenty-two SAMP8 mice and divided them into two groups (9–11 mice/group): taurine group and control group (DW). At 42 weeks of age, five mice in each group were anaesthetized using 2% sodium phenobarbital intraperitoneal injection—works by depressing the activity of brain and central nervous system—and then mice were sacrificed and transcardially perfused with saline. There was no significant difference in body weight between the taurine and DW groups (29.7 ± 2.3 and 29.0 ± 2.9 g, respectively at 42 weeks-old).

### Brain tissue preparation

To prepare tissue samples, the whole brain tissue was extracted and separated from the center. Right side of the brain was post-fixed in 4% paraformaldehyde (PFA) for immunohistochemistry. The hippocampus and cortex were quickly dissected from the left side of the brain and immediately placed on dry ice. After being quickly frozen using dry ice, samples were stored at − 80 °C until use. All dissections were performed on ice.

### Immunohistochemistry

Formalin-fixed mouse brain tissues were kept in phosphate-buffered saline (PBS) for one day. Subsequently, brain tissue was embedded into a paraffin block, which was then sectioned into 5-µm thick slices using Leica Microsystem (Wetzler, Germany) following a routine protocol. The sections were incubated at 45 °C for 48 h in an incubator. Paraffin-embedded brain sections were rehydrated in a series of xylene and ethanol. Tissue sections were then boiled for 5 min in 5% urea using a microwave at 500 W for antigen retrieval and then treated with 1% H_2_O_2_ for 20 min at room temperature to block endogenous peroxidase. Tissue sections were then blocked with 1% skim milk in PBS (pH 7.4) for 25 min. Sections were then incubated overnight with primary antibody anti-taurine (rabbit IgG, produced by Ma et al., as previously reported^50^), anti-iba1 (rabbit IgG, Fujifilm Wako Pure Chemical Corporation, 013-27691), anti-TNF-alpha (rabbit IgG, Abcam, ab6671), anti-tau (phospho S396) (rabbit IgG, Abcam, ab109390), anti-NeuN (rabbit IgG, Abcam, ab177487) and anti-beta-amyloid (rabbit IgG, Abcam, ab201060) in a humid chamber. After washing, the sections were treated with a specific biotinylated secondary antibody and avidin–biotin peroxidase conjugate (ABC kit; Vector Laboratories, Burlingame, CA, USA), according to the manufacturer’s protocol. Immunoreactions were visualized by incubation with a DAB (brown) peroxidase substrate kit (Nacalai Tesque, Kyoto, Japan). In some cases, the nuclei were counterstained with hematoxylin and dehydrated before being mounted in malinol (Muto Pure Chemical Co., Ltd. Tokyo). For each antibody, we included a positive control slide to confirm the staining intensity.

### Quantification

Images were captured using a microscope (BX53, Olympus). The number of activated microglia, TNF-alpha-positive cells, amyloid plaques and NeuN-positive cells were counted manually. Staining intensity and positive areas of taurine, TNF-alpha, phospho-tau and amyloid-beta were quantified blindly in the experimental groups. For the hippocampal analyses, the entire hippocampal field, including CA1 and other parts, of a mouse was considered, and for the cortical analysis, a specific area of the temporal cerebral cortex of a mouse was considered. For each antibody 3–5 samples were collected from each group. Images from each group were analyzed using the ImageJ software (version 1.5, Wayne Rasband, NIH, USA), and a threshold level was applied to each image to select and measure the total amount of specific immunostaining. The threshold level settings for each antibody were the same for all groups. The staining intensity was measured as the proportion (%) of the stained area compared to the total area of the image.

### Western blot analysis from brain tissue

The hippocampus and the cortex were separated using disposable blades. The whole hippocampus and cortex were individually homogenized in ice-cold cell lysis RIPA buffer (Cell Signaling Technology, Danvers, MA, USA) and 1 × protease inhibitor (Nacalai Tesque). The homogenates were then sonicated and centrifuged at 14,000*g* for 15 min at 4 °C. The supernatant was collected, and the total protein concentration was measured using a BCA protein assay kit (Pierce Chemical, Rockford, IL, USA). The samples were prepared in a loading buffer and boiled for 10 min at 70 °C. Equal amounts of protein were loaded onto 5–20% SDS-PAGE (SuperSep Ace, Wako Pure Chemical Industries, Osaka, Japan) gels and transferred to PVDF membranes. The membranes were then blocked with 5% skim milk for 1 h in Tris-buffered saline containing 0.1% Tween 20 (TBST) at room temperature. The PVDF membranes were incubated with primary antibody anti-TAUT (mouse IgG, Santa Cruz Biotechnology Inc., sc-393036), anti-PHF1 (rabbit IgG, Abcam, ab184951), anti-tau (phospho S396) (rabbit IgG, Abcam, ab109390), anti-tau (rabbit IgG, Abcam, ab76128), anti-NeuN (rabbit IgG Abcam, ab177487) and anti-TREM2 (rabbit IgG, ThermoFisher, PA5-119690) overnight at 4 °C. After washing with TBST, the membranes were incubated with horseradish peroxidase-conjugated secondary antibodies for 30 min at room temperature. The membrane was visualized using enhanced chemiluminescence detection reagent (GE Healthcare, CA, United States) and detected using Amersham ImageQuant 800 (Fujifilm, Tokyo, Japan). The membranes were then stripped using a stripping buffer (Thermo Scientific, 46430) and re-incubated with anti-beta-actin (rabbit IgG, Cell Signaling Technology, 4967S) for normalization. The obtained signal densities were quantified using the ImageJ software. The image was converted to 8-bit by opening an image with ImageJ software. By using the rectangular selection tool first lane were drawn and selected, then all the remaining lanes are drawn in the same way by dragging the selection tool. After that the plot was drawn for analysis. Then the area under the sharp peak for all lanes was marked by using straight lines from tool menu. Background peaks were reduced with straight line where necessary. Then all band peaks were selected by wand tool. Image of internal control (beta-actin) was analyzed by similar process for normalization.

### Statistical analysis

The quantitative results are presented as the means ± SD. All statistical results were analyzed using Student’s t-tests to determine statistical significance. *P* values less than 0.05 were considered to be statistically significant. All data are presented as means ± SD (bar), and asterisks in the graphs indicate the following: *p < 0.05, **p < 0.01, ***p < 0.001 and ns (not significant) (Supplementary Information S1).

### Supplementary Information

Supplementary Figures.

## Data Availability

The data and materials in the current study are available from the corresponding author (M.M.) on reasonable request.
