# Supplementary material for: Taurine reduces microglia activation in the brain of aged senescence-accelerated mice by increasing the level of TREM2
Source: Sci Rep. 2024 Mar 28;14:7427. doi: 10.1038/s41598-024-57973-4 (PMC10978912; doi:10.1038/s41598-024-57973-4)
Supplement: Supplementary file 1 — Supplementary Figures. [file 41598_2024_57973_MOESM1_ESM.pptx]

## Slide 1
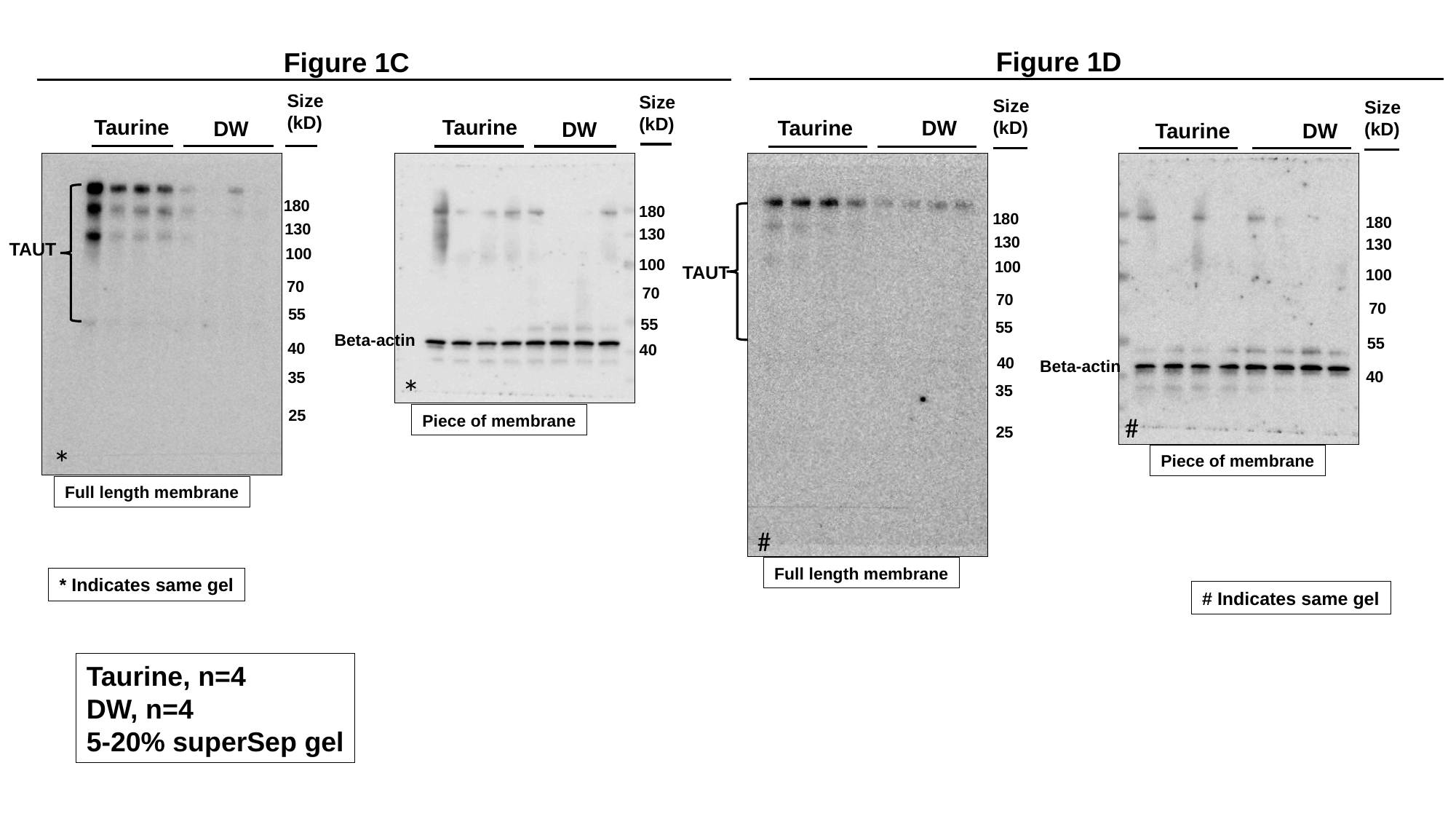

Figure 1D
Figure 1C
Size
(kD)
Size
(kD)
Size
(kD)
Size
(kD)
Taurine
Taurine
Taurine
DW
DW
DW
Taurine
DW
180
180
180
180
130
130
130
130
 TAUT
100
100
100
 TAUT
100
70
70
70
70
55
55
55
 Beta-actin
55
40
40
40
 Beta-actin
40
35
*
35
25
Piece of membrane
#
25
*
Piece of membrane
Full length membrane
#
Full length membrane
* Indicates same gel
# Indicates same gel
Taurine, n=4
DW, n=4
5-20% superSep gel

## Slide 2
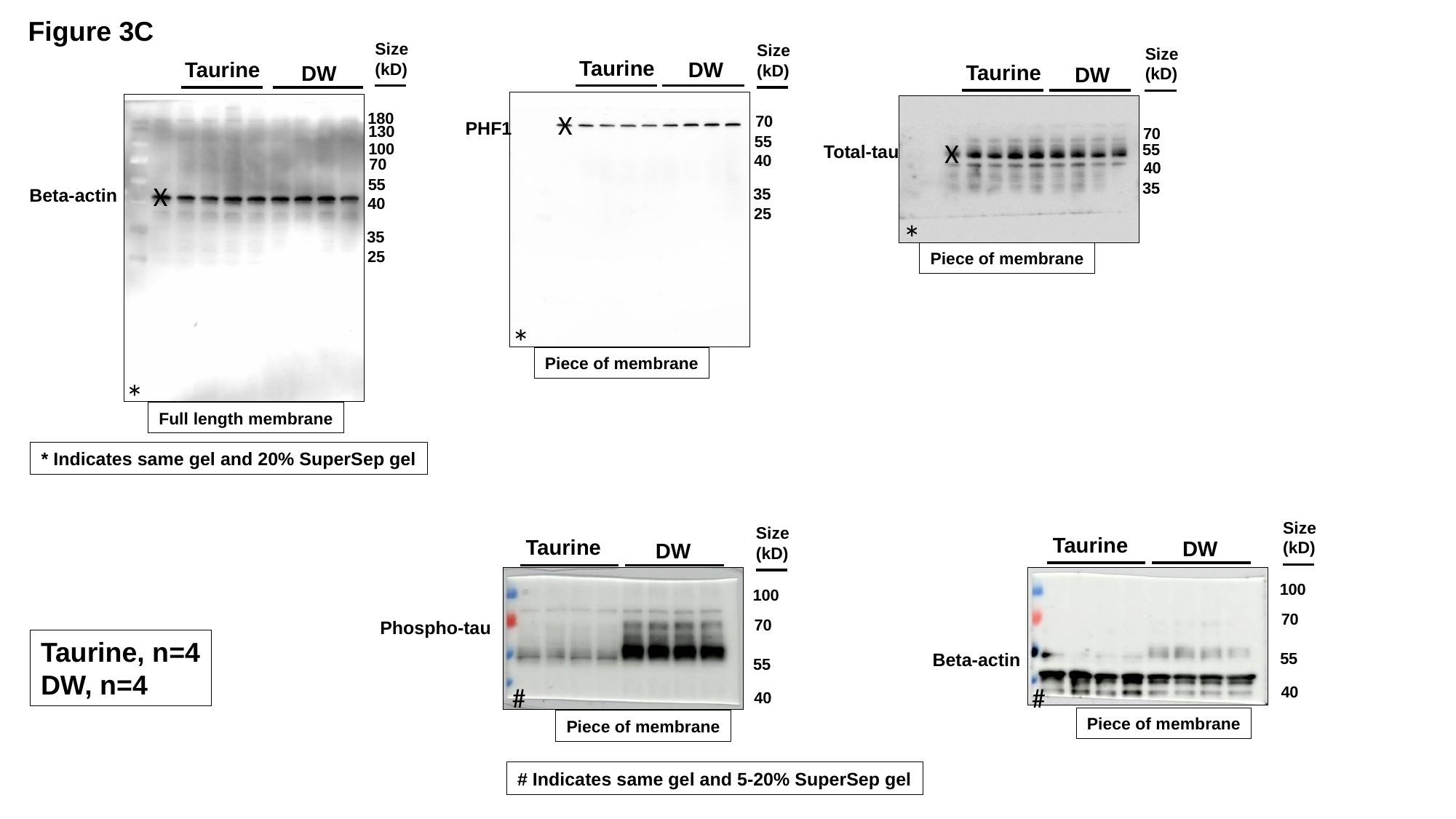

Figure 3C
Size
(kD)
Size
(kD)
Size
(kD)
Taurine
Taurine
DW
Taurine
DW
DW
180
X
70
PHF1
130
70
55
X
100
55
Total-tau
40
70
40
55
35
X
Beta-actin
35
40
25
*
35
25
Piece of membrane
*
Piece of membrane
*
Full length membrane
* Indicates same gel and 20% SuperSep gel
Size
(kD)
Size
(kD)
Taurine
Taurine
DW
DW
100
100
70
70
Phospho-tau
Taurine, n=4
DW, n=4
Beta-actin
55
55
#
#
40
40
Piece of membrane
Piece of membrane
# Indicates same gel and 5-20% SuperSep gel

## Slide 3
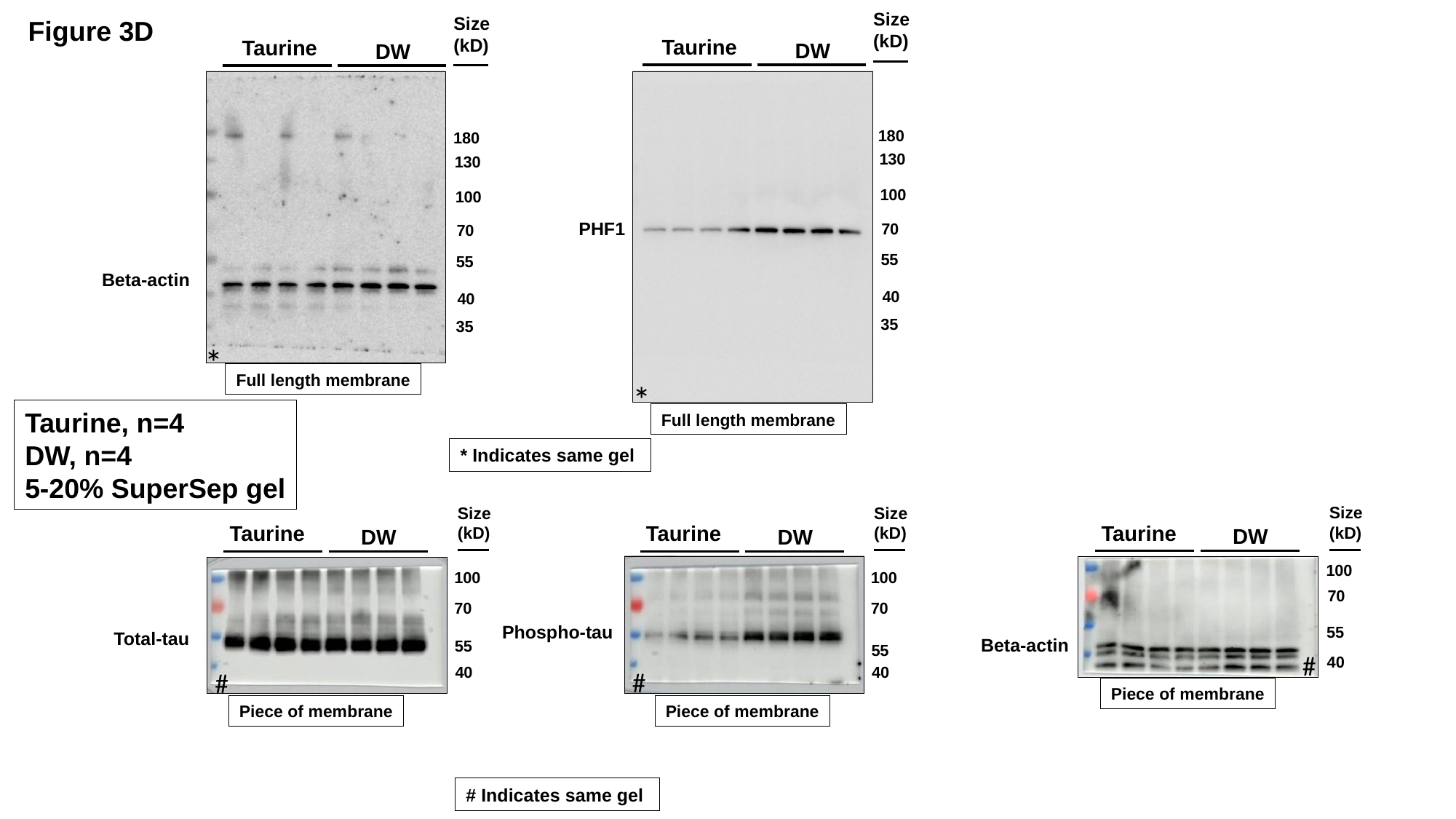

Size
(kD)
Size
(kD)
Figure 3D
Taurine
Taurine
DW
DW
180
180
130
130
100
100
PHF1
70
70
55
55
Beta-actin
40
40
35
35
*
Full length membrane
*
Taurine, n=4
DW, n=4
5-20% SuperSep gel
Full length membrane
* Indicates same gel
Size
(kD)
Size
(kD)
Size
(kD)
Taurine
Taurine
Taurine
DW
DW
DW
100
100
100
70
70
70
Phospho-tau
55
Total-tau
Beta-actin
55
55
#
40
40
40
#
#
Piece of membrane
Piece of membrane
Piece of membrane
# Indicates same gel

## Slide 4
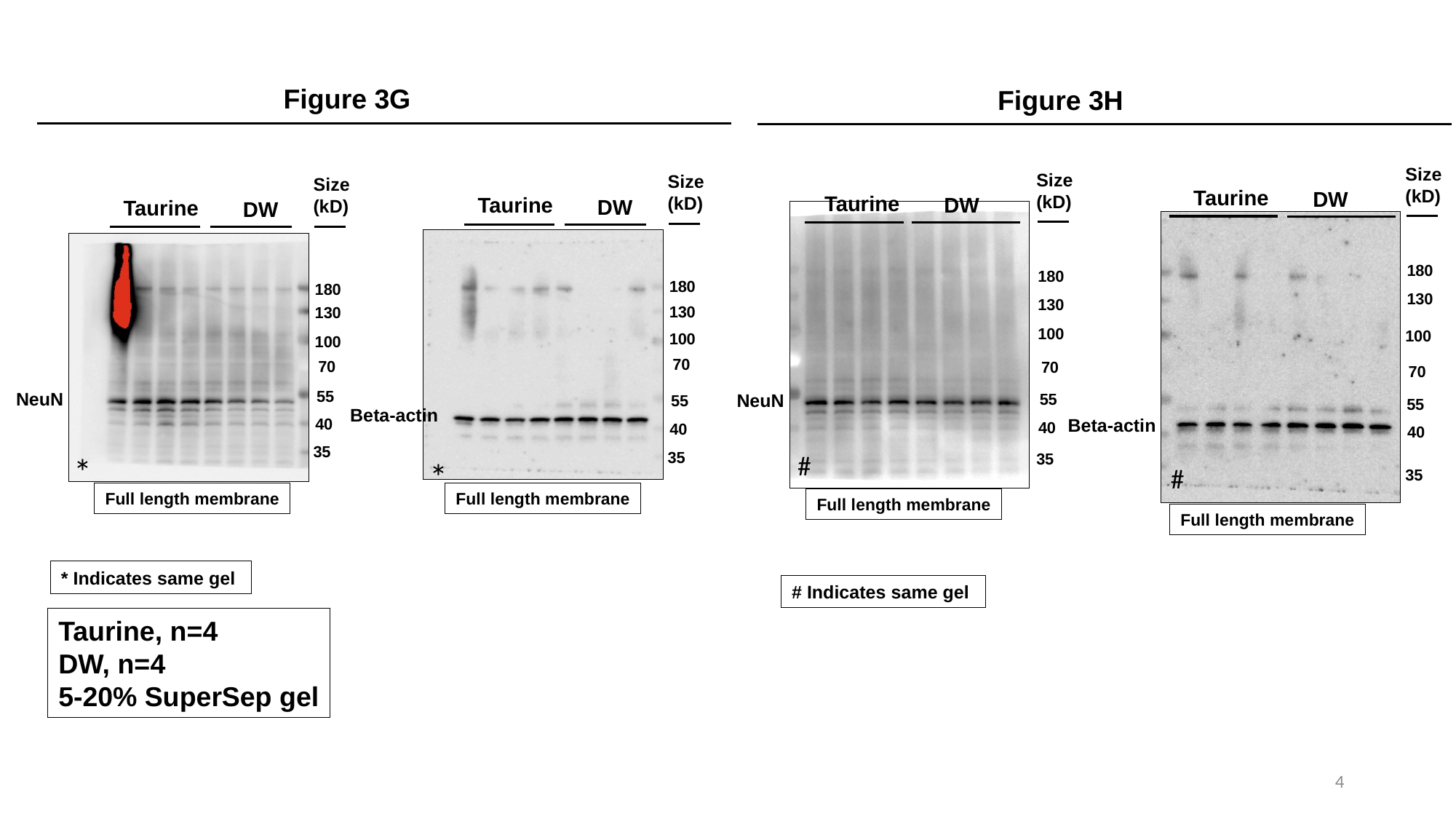

Figure 3G
Figure 3H
Size
(kD)
Size
(kD)
Size
(kD)
Size
(kD)
Taurine
DW
Taurine
DW
Taurine
DW
Taurine
DW
180
180
180
180
130
130
130
130
100
100
100
100
70
70
70
70
55
NeuN
55
NeuN
55
55
Beta-actin
40
Beta-actin
40
40
40
35
35
*
#
35
*
#
35
Full length membrane
Full length membrane
Full length membrane
Full length membrane
* Indicates same gel
# Indicates same gel
Taurine, n=4
DW, n=4
5-20% SuperSep gel
4

## Slide 5
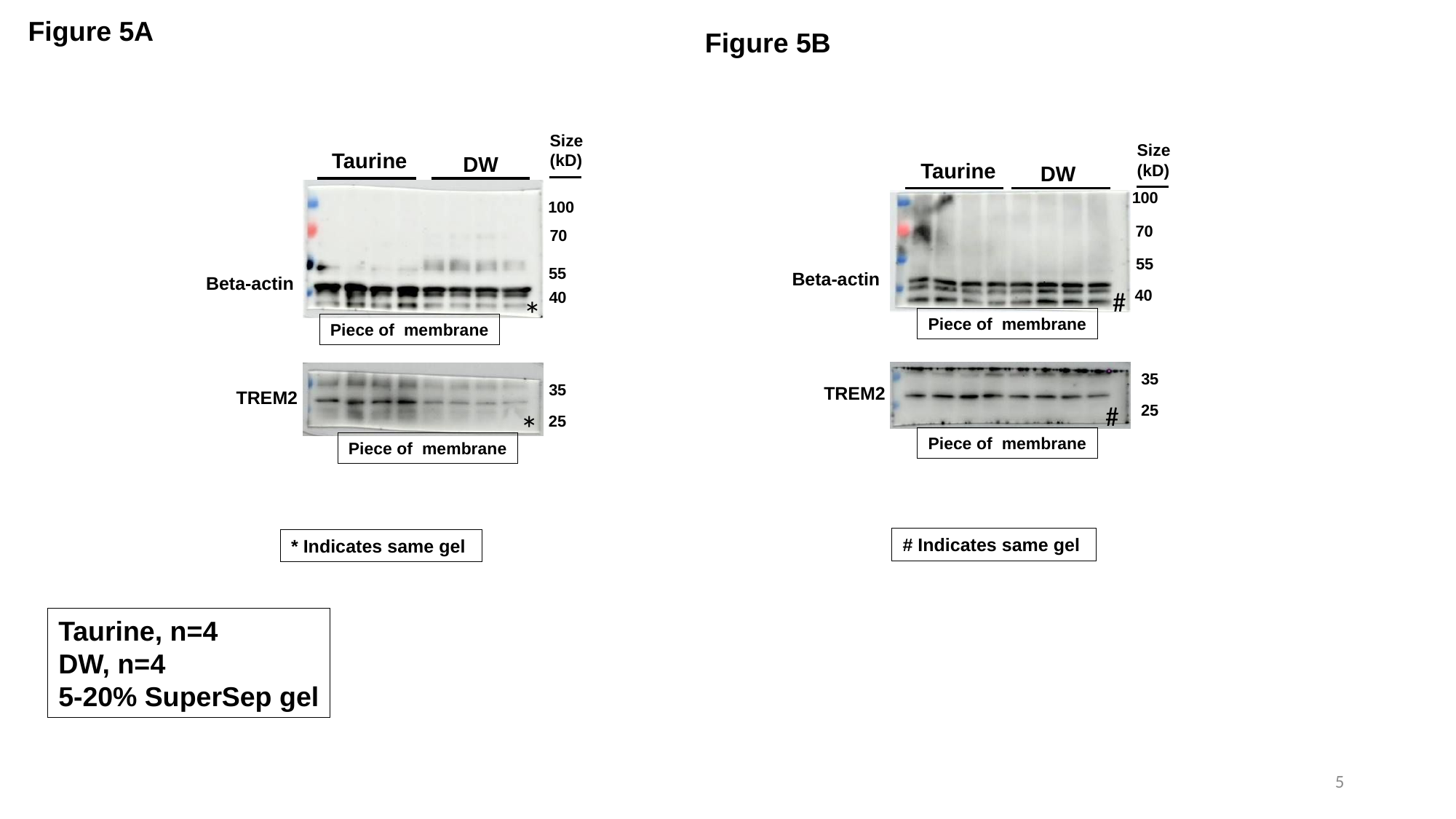

Figure 5A
Figure 5B
Size
(kD)
Size
(kD)
Taurine
DW
Taurine
DW
100
100
70
70
55
55
Beta-actin
Beta-actin
#
40
40
*
Piece of membrane
Piece of membrane
35
35
TREM2
TREM2
#
25
*
25
Piece of membrane
Piece of membrane
# Indicates same gel
* Indicates same gel
Taurine, n=4
DW, n=4
5-20% SuperSep gel
5
